# Factors associated with delirium among survivors of acute respiratory distress syndrome: a nationwide cohort study

**DOI:** 10.1186/s12890-021-01714-0

**Published:** 2021-11-01

**Authors:** Tak Kyu Oh, Hye Youn Park, In-Ae Song

**Affiliations:** 1 Department of Anesthesiology and Pain Medicine, Seoul National University Bundang Hospital, Gumi-ro, 173, Beon-gil, Bundang-gu, Seongnam, 13620 South Korea; 2 Department of Anesthesiology and Pain Medicine, College of Medicine, Seoul National University, Seoul, South Korea; 3 Department of Psychiatry, Seoul National University Bundang Hospital, Seongnam, South Korea

**Keywords:** Critical Care, Delirium, Mortality, Respiratory distress syndrome

## Abstract

**Background:**

The prevalence of delirium, its associated factors, and its impact on long-term mortality among survivors of acute respiratory distress syndrome (ARDS) is unclear.

**Methods:**

Since this was a population-based study, data were extracted from the National Health Insurance database in South Korea. All adults who were admitted to intensive care units with a diagnosis of ARDS between January 1, 2010, and December 31, 2019, and who survived for ≥ 60 days were included. The International Statistical Classification of Diseases and Related Health Problems, tenth revision code of delirium (F05) was used to extract delirium cases during hospitalization.

**Results:**

A total of 6809 ARDS survivors were included in the analysis, and 319 patients (4.7%) were diagnosed with delirium during hospitalization. In the multivariable logistic regression analysis after covariate adjustment, male sex (odds ratio [OR] 1.60, 95% confidence interval [CI] 1.23, 2.08; *P* < 0.001), longer duration of hospitalization (OR 1.02, 95% CI 1.01, 1.03; *P* < 0.001), neuromuscular blockade use (OR 1.50, 95% CI 1.12, 2.01; *P* = 0.006), benzodiazepine (OR 1.55, 95% CI 1.13, 2.13; *P* = 0.007) and propofol (OR 1.48, 95% CI 1.01, 2.17; *P* = 0.046) continuous infusion, and concurrent depression (OR 1.31, 95% CI 1.01, 1.71; *P* = 0.044) were associated with a higher prevalence of delirium among ARDS survivors. In the multivariable Cox regression analysis after adjustment for covariates, the occurrence of delirium was not significantly associated with 1-year all-cause mortality, when compared to the other survivors who did not develop delirium (hazard ratio: 0.85, 95% CI 1.01, 1.71; *P* = 0.044).

**Conclusions:**

In South Korea, 4.7% of ARDS survivors were diagnosed with delirium during hospitalization in South Korea. Some factors were potential risk factors for the development of delirium, but the occurrence of delirium might not affect 1-year all-cause mortality among ARDS survivors.

**Supplementary Information:**

The online version contains supplementary material available at 10.1186/s12890-021-01714-0.

## Background

Delirium is a serious disturbance in mental abilities that results in an acute confusional status and deteriorating awareness of the environment [1]. It is commonly diagnosed using the diagnostic and statistical manual of mental disorders, fifth edition (DSM-5) criteria [2]. It occurs frequently among critically ill patients admitted in the intensive care unit (ICU) [3]. Since delirium is a significant risk factor of prolongation of ICU stay and poorer survival outcomes up to one year after discharge among ICU patients [4, 5], effective prevention and management of delirium are important.

For critically ill patients in the ICU, the reported prevalence of delirium is 25–31.8% [5, 6]. In particular, critically ill patients who undergo mechanical ventilatory support have a higher risk of delirium; this is an independent predictor of higher 6-month mortality [7, 8]. Acute respiratory distress syndrome (ARDS) is characterized by refractory hypoxemia from respiratory failure [9]. Thus, most patients with ARDS require mechanical ventilation, and 36% of patients requiring mechanical ventilation experienced agitation under light sedation [10]. In addition, ARDS is associated with a higher risk of delirium during ICU stay [11]. However, studies on the relationship between delirium, risk factors, and mortality of patients with ARDS are few. A recent retrospective cohort study reported that 43% (124/286) of patients with ARDS were diagnosed with ICU delirium [12]. In that study, the impact of delirium on mortality of patients with ARDS was not assessed, and the sample size was relatively small [12]. Moreover, the prevalence and impact of delirium on long-term mortality during hospitalization among ARDS survivors have not yet been identified.

Therefore, using a nationwide claim database in South Korea, this study aimed to investigate the prevalence and factors associated with the occurrence of delirium among ARDS survivors. In addition, we examined whether the occurrence of delirium during hospitalization was associated with long-term mortality.

## Methods

### Study design and ethical statement

This study is a nationwide population-based cohort study. It was conducted in accordance with the Strengthening of the Reporting of Observational Studies in Epidemiology guidelines. The study protocol was approved by the Institutional Review Board (IRB) (X-2008-630-903), and the National Health Insurance Service (NHIS) permitted data sharing after approval of the study protocol (NHIS-2021-1-424). The requirement of informed consent was waived by the IRB because anonymized data was used in this study.

### Data source and study population

Data (demographic, socioeconomic, and treatment data of all Korean individuals in the sole and public health insurance system in South Korea) was obtained from the NHIS database. All diagnoses of diseases had to be registered by physicians using the International Statistical Classification of Diseases and Related Health Problems, Tenth revision (ICD-10) codes in the NHIS database. The prescription information of all procedures and/or drugs also had to be registered in the NHIS database. These registrations enable patients to receive financial support for treatment expenses by the government.

All adult patients (aged ≥ 18 years) who were admitted to the ICU for ARDS (J80) between January 1, 2010, and December 31, 2019, were eligible. Patients with a main diagnosis or secondary diagnosis of ARDS were included because ARDS is a clinical syndrome that may occur concurrently with other main diseases such as pneumonia, sepsis, or pancreatitis [13]. The main diagnosis of each patient was determined by the NHIS after hospital discharge or death, as a disease requiring close follow-up or emergency treatment during the patient’s hospitalization. If a patient was admitted to the ICU with a diagnosis of ARDS ≥ 2 times during the study period, only the first ICU admission was considered in this study. To focus on delirium during hospitalization, patients with ARDS who died within 60 days after hospitalization were excluded because 60-day mortality is a common primary endpoint for patients with severe ARDS [14]. Therefore, patients with ARDS who survived ≥ 60 days after the diagnosis of ARDS were considered ARDS survivors and included in the final analysis.

### Endpoints and outcomes

The primary endpoint was the diagnosis of delirium during hospitalization and was evaluated from the date of ARDS diagnosis to 60 days after ARDS diagnosis. The ICD-10 code, F05, was used to extract delirium diagnoses. In South Korea, DSM-5 criteria isusually used for diagnosis of delirium in ICU [2], and the registration of ICD-10 codes of delirium enable the financial support of treatment. Moreover, most medical staff in Korean ICUs used Korean version of confusion assessment method for the ICU (CAM-ICU) routinely for screening and assessment of delirium during ICU stays [15]. The ARDS survivors who were diagnosed with delirium constituted the delirium group, while the remaining patients constituted the control group. The secondary endpoint was 1-year all-cause mortality, which was evaluated from the date of ARDS diagnosis to one year after ARDS diagnosis. To better follow up the patients with ARDS for at least one year after ARDS diagnosis, the exact date of death was extracted until December 31, 2020.

First, the prevalence of delirium among ARDS survivors and the factors associated with delirium were investigated. Second, the association between development of delirium during hospitalization among ARDS survivors and 1-year all-cause mortality was examined.

### Covariates

The following variables were considered covariates in this study: age and sex were demographic variables. Household income level and employment status at the time of ARDS treatment was included to reflect patients’ socioeconomic status. The patients were divided into four groups according to household income levels using quartile ratios. Household income level was registered annually in the NHIS database to determine insurance premiums in South Korea. The admitting department was included as a covariate and classified into two groups (internal medicine and non-internal medicine). Length of hospitalization (day) and total cost of hospitalization (United States dollars) were covariates. Since higher case volume centers are related to better survival outcomes of patients with ARDS [16], the annual case volume of ICU admission for ARDS treatment was considered a covariate. All patients with ARDS were divided into four groups using quartile ratios, based on the hospital in which they were admitted for ARDS treatment: Q1 ≤ 4; Q2, 5–14; Q3, 15–28; and Q4 ≥ 28. A main diagnosis of ARDS was considered as a covariate, and diagnoses of shock (R57) or sepsis (A40, A41, and R65.2) were also considered as covariates. If a patient with ARDS had a main diagnosis of sepsis and a secondary diagnosis of ARDS, the diagnosis was sepsis-associated ARDS. To reflect the comorbidity status of patients with ARDS, the Charlson comorbidity index (CCI) was calculated using registered ICD-10 codes within one year before the date of ARDS diagnosis (Additional file 1: Table S1). All patients were divided into four groups according to their CCI scores (0–1, 2–3, 4–5, and ≥ 6). Concurrent psychiatric illnesses such as depression (F32, F33, and F34.1), anxiety disorder (F40 and F 41), post-traumatic stress disorder (F43.1), alcohol abuse (F10), and other substance abuse (F11–19) were considered because of the close relationship between concurrent psychiatric disorders and development of delirium among hospitalized patients [17, 18]. Moreover, underlying disability at admission for ARDS was extracted and considered as a covariate. In South Korea, all disabilities should be registered in the NHIS database to receive various benefits under social welfare programs. The disabilities were divided into two groups such as brain disability and non-brain disability, because delirium is common in patients with neurological disease [19]. Regarding treatment, extracorporeal membrane oxygenation support, neuromuscular blockade use, continuous renal replacement therapy use, duration of mechanical ventilation (day), and cardiopulmonary resuscitation experience were considered as covariates. Information regarding benzodiazepine continuous infusion (midazolam, diazepam, and lorazepam), opioid continuous infusion, and propofol infusion were collected as covariates. Admission to isolated ICU was also collected and considered as a covariate, because isolation in ICU was known as environmental risk factor of delirium in ICU [20].

### Statistical analysis

The clinicopathological characteristics of ARDS survivors are presented as means with standard deviations (SDs) for continuous variables and numbers with percentages for categorical variables. The t-test was used to compare continuous variables, and the chi-square test was used to compare categorical variables between the delirium and control groups. We fitted a multivariable logistic regression model for diagnosing delirium among ARDS survivors. All covariates were included in the model for multivariable adjustment. The Hosmer–Lemeshow test was performed to confirm the goodness of fit of the multivariable model. The results of the logistic regression analysis are presented as odds ratios (ORs) with 95% confidence intervals (CIs). A multivariable Cox regression model for 1-year all-cause mortality among ARDS survivors was constructed. All covariates were included in the adjusted model. The log–log plot was used to confirm that the central assumption of the Cox proportional hazard model was satisfied. The results of the Cox regression model are presented as hazard ratios (HRs) with 95% CIs. There was no multicollinearity in both multivariable logistic and Cox regression models as criteria of variance inflation factors: < 2.0 between all variables. All statistical analyses were performed using R software (version 4.0.3, R packages, R Project for Statistical Computing, Vienna, Austria). *P* values < 0.05 were considered statistically significant for all analyses.

## Results

**Fig. 1:**
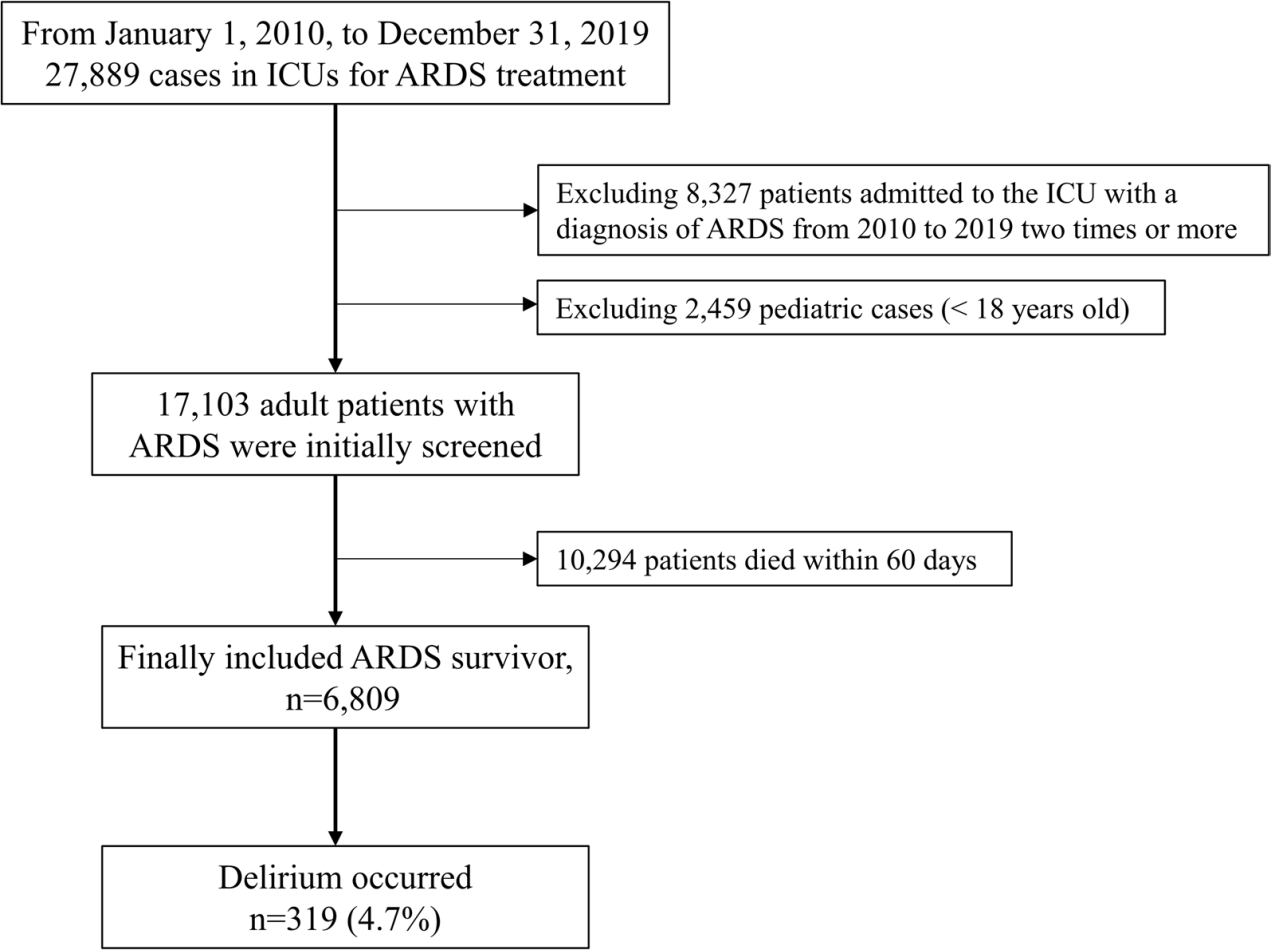
Selection process flow chart of acute respiratory distress syndrome survivors. ARDS, acute respiratory distress syndrome

A total of 27,889 patients were admitted in the ICU and diagnosed with ARDS in South Korea. After excluding 8,327 patients with ≥ 2 ICU admissions and 2,459 pediatric patients (below 18 years old), 17,103 adult patients with ARDS were screened initially. Among them, 10, 294 (60.2%) patients who died within 60 days after ARDS diagnosis were excluded, and 6,809 ARDS survivors were finally included in the analysis. Among them, 319 (4.7%) patients were diagnosed with delirium during hospitalization (Fig. 1).

The clinicopathological characteristics of the ARDS survivors are presented in Table 1. The mean age of the ARDS survivors was 66 (SD: 17.2) years, and 61.4% (4180/6809) of the patients were men. The results of the comparison of clinicopathological characteristics between the delirium and control groups are presented in Table 2. The mean length of hospital stay of the delirium group was longer than that of the control group (30.2 [SD: 21.3] vs. 20.2 [SD: 16.8] days, respectively; *P* < 0.001). The mean duration of mechanical ventilation in the delirium group was also longer than that in the control group (6.5 [SD: 9.2] vs. 4.5 [SD: 8.8] days, respectively; *P* < 0.001). The proportion of patients with concurrent depression was higher in the delirium group (34.2% [109/319]) than in the control group (27.1% [1757/6490], *P* = 0.006).

**Table 1:** The clinicopathological characteristics of the ARDS survivors

Variable	Mean (SD) or N (%)
Age, year	66.0 (17.2)
Sex, male	4180 (61.4)
Having a job at admission for ARDS	3555 (52.2)
Annual income level at ARDS treatment	
Q1 (Lowest)	2031 (29.8)
Q2	1073 (15.8)
Q3	1333 (19.6)
Q4 (Highest)	2227 (32.7)
Unknown	145 (2.1)
Admitting department	
IM	5480 (80.5)
Non-IM	1329 (19.5)
Length of hospitalization, day	20.7 (17.2)
Total cost for hospitalization, USD	10,799.2 (13,605.4)
Annual case volume of ARDS admission	
Q1 ≤ 4	1417 (20.8)
Q2: 5–14	1648 (24.2)
Q3: 15–28	1966 (28.9)
Q4 ≥ 28	1778 (26.1)
Main diagnosis of ARDS	3390 (49.8)
Sepsis associated ARDS	393 (5.3)
Diagnosis of shock during hospitalization	363 (5.3)
Underlying brain disability	484 (7.1)
Underlying non-brain disability	1480 (21.7)
Isolated ICU admission	196 (2.9)
CCI at hospital admission for ARDS	
0–1	1523 (22.4)
2–3	2171 (31.9)
4–5	1558 (22.9)
≥ 6	1557 (22.9)
ECMO support	317 (4.7)
NMB use	2238 (32.9)
CRRT use	299 (4.4)
Benzodiazepine continuous infusion	3889 (57.1)
Opioid continuous infusion	1049 (15.4)
Propofol continuous infusion	560 (8.2)
Duration of Mechanical Ventilator use, day	4.6 (8.8)
Experience of CPR during hospitalization	236 (3.5)
Concurrent other psychiatric illness	
Depression	1866 (27.4)
Anxiety disorder	2324 (34.1)
PTSD	11 (0.2)
Alcohol abuse	314 (4.6)
Other substance abuse	36 (0.5)
Year of admission for ARDS	
2010	849 (12.5)
2011	656 (9.6)
2012	607 (8.9)
2013	527 (7.7)
2014	641 (9.4)
2015	621 (9.1)
2016	831 (12.2)
2017	705 (10.4)
2018	723 (10.6)
2019	649 (9.5)
1-year all-cause mortality	1868 (27.4)

ARDS, acute respiratory distress syndrome; SD, standard deviation; IM, internal medicine; USD, United States Dollar; CCI, Charlson comorbidity index; ECMO, extracorporeal membrane oxygenation; NMB, neuromuscular blockade; CRRT, continuous renal replacement therapy; CPR, cardiopulmonary resuscitation; PTSD, post-traumatic stress disorder

**Table 2:** The comparison of clinicopathological characteristics between the delirium and control groups

Variable	Delirium group *n* = 319	Control group *n* = 6,490	*P* value
Age, year	66.7 (17.3)	66.0 (17.2)	0.414
Sex, male	232 (72.7)	3948 (60.8)	< 0.001
Having a job at admission for ARDS	175 (54.9)	3380 (52.1)	0.332
Annual income level at ARDS treatment			0.510
Q1 (Lowest)	92 (28.8)	1939 (29.9)	
Q2	50 (15.7)	1023 (15.8)	
Q3	70 (21.9)	1263 (19.5)	
Q4 (Highest)	104 (32.6)	2123 (32.7)	
Unknown	3 (0.9)	142 (2.2)	
Admitting department: IM	273 (85.6)	5207 (80.2)	0.019
Length of hospitalization, day	30.2 (21.3)	20.2 (16.8)	< 0.001
Total cost for hospitalization, USD	18,394.5 (17,772.2)	10,425.9 (13,257.2)	< 0.001
Underlying brain disability	14 (4.4)	470 (7.2)	0.068
Underlying non-brain disability	74 (23.2)	1406 (21.7)	0.517
Annual case volume of ARDS admission			< 0.0012
Q1 ≤ 4	38 (11.9)	1379 (21.2)	
Q2: 5–14	64 (20.1)	1584 (24.4)	
Q3: 15–28	100 (31.3)	1866 (28.8)	
Q4 ≥ 28	117 (36.7)	1661 (25.6)	
Main diagnosis of ARDS	162 (50.8)	3228 (49.7)	0.715
Sepsis associated ARDS	37 (11.6)	796 (12.3)	0.723
Diagnosis of shock during hospitalization	23 (7.2)	340 (5.2)	0.126
Isolated ICU admission	13 (4.1)	183 (2.8)	0.190
CCI at hospital admission for ARDS			0.584
0–1	62 (19.4)	1461 (22.5)	
2–3	102 (32.0)	2069 (31.9)	
4–5	76 (23.8)	1482 (22.8)	
≥ 6	79 (24.8)	1478 (22.8)	
ECMO support	23 (7.2)	294 (4.5)	0.027
NMBA use	171 (53.6)	2067 (31.8)	< 0.001
CRRT use	20 (6.3)	279 (4.3)	0.094
Benzodiazepine continuous infusion	248 (77.7)	3641 (56.1)	< 0.001
Opioid continuous infusion	77 (24.1)	972 (15.0)	< 0.001
Propofol continuous infusion	51 (16.0)	509 (7.8)	< 0.001
Duration of Mechanical Ventilator use, day	6.5 (9.2)	4.5 (8.8)	< 0.001
Experience of CPR during hospitalization	16 (5.0)	220 (3.4)	0.121
Concurrent depression	109 (34.2)	1757 (27.1)	0.006
Concurrent anxiety disorder	119 (37.3)	2205 (34.0)	0.221
Concurrent PTSD	1 (0.3)	10 (0.2)	0.489
Concurrent alcohol abuse			
Concurrent other substance abuse			
Year of admission for ARDS			0.099
2010	28 (8.8)	821 (12.7)	
2011	19 (6.0)	637 (9.8)	
2012	29 (9.1)	578 (8.9)	
2013	34 (10.7)	493 (7.6)	
2014	33 (10.3)	608 (9.4)	
2015	34 (10.7)	587 (9.0)	
2016	43 (13.5)	788 (12.1)	
2017	32 (10.0)	673 (10.4)	
2018	39 (12.2)	684 (10.54)	
2019	28 (8.8)	621 (9.6)	
1-year all-cause mortality	92 (28.8)	1776 (27.4)	0.564

Presented as means with standard deviations for continuous variables and numbers with percentages for categorical variables

ARDS, acute respiratory distress syndrome; IM, internal medicine; USD, United States Dollar; CCI, Charlson comorbidity index; ECMO, extracorporeal membrane oxygenation; NMB, neuromuscular blockade; CRRT, continuous renal replacement therapy; CPR, cardiopulmonary resuscitation; PTSD, post-traumatic stress disorder

Table 3 shows the results of the multivariable logistic regression analysis of the occurrence of delirium among ARDS survivors. Male sex (OR 1.60, 95% CI 1.23, 2.08; *P* < 0.001), longer duration of hospitalization (OR 1.02, 95% CI 1.01, 1.03; *P* < 0.001), neuromuscular blockade use (OR 1.50, 95% CI 1.12, 2.01; *P* = 0.006), benzodiazepine (OR 1.55, 95% CI 1.13, 2.13; *P* = 0.007) and propofol (OR 1.48, 95% CI 1.01, 2.17; *P* = 0.046) continuous infusion, and concurrent depression (OR 1.31, 95% CI 1.01, 1.71; *P* = 0.044) were associated with a higher prevalence of delirium among ARDS survivors.

**Table 3:** Multivariable logistic regression analysis of the occurrence of delirium among ARDS survivors

Variable	OR (95% CI)	*P* value
Age, year	1.01 (1.00, 1.02)	0.072
Sex, male (vs female)	1.60 (1.23, 2.08)	< 0.001
Having a job at admission for ARDS	1.08 (0.85, 1.38)	0.511
Annual income level at ARDS treatment		
Q2 (vs Q1: Lowest)	0.95 (0.66, 1.38)	0.795
Q3 (vs Q1: Lowest)	1.09 (0.78, 1.52)	0.629
Q4: Highest (vs Q1: Lowest)	0.86 (0.63, 1.17)	0.344
Unknown (vs Q1: Lowest)	0.46 (0.14, 1.49)	0.195
Admitting department: IM (vs non-IM)	1.44 (1.02, 2.02)	0.037
Length of hospitalization, day	1.02 (1.01, 1.03)	< 0.001
Total cost for hospitalization, 1000 USD	1.01 (0.99, 1.02)	0.333
Underlying brain disability	0.65 (0.37, 1.14)	0.129
Underlying non-brain disability	0.99 (0.74, 1.31)	0.924
Annual case volume of ARDS admission		
Q2: 5–14 (vs Q1 ≤ 4)	1.06 (0.69, 1.61)	0.805
Q3: 15–28 (vs Q1 ≤ 4)	1.32 (0.88, 1.98)	0.176
Q4 ≥ 28 (vs Q1 ≤ 4)	1.79 (1.20, 2.69)	0.005
Main diagnosis of ARDS (vs Secondary diagnosis of ARDS)	0.94 (0.74, 1.19)	0.609
Sepsis associated ARDS	0.67 (0.46, 0.96)	0.030
Diagnosis of shock during hospitalization	1.02 (0.65, 1.61)	0.927
Isolated ICU admission	0.99 (0.53, 1.85)	0.981
CCI at hospital admission for ARDS		
2–3 (vs 0–1)	1.01 (0.72, 1.41)	0.961
4–5 (vs 0–1)	0.96 (0.67, 1.38)	0.830
≥ 6 (vs 0–1)	0.95 (0.66, 1.36)	0.772
ECMO support	0.98 (0.57, 1.67)	0.930
NMB use	1.50 (1.12, 2.01)	0.006
CRRT use	0.84 (0.50, 1.40)	0.503
Benzodiazepine continuous infusion	1.55 (1.13, 2.13)	0.007
Opioid continuous infusion	1.05 (0.73, 1.53)	0.781
Propofol continuous infusion	1.48 (1.01, 2.17)	0.046
Duration of Mechanical Ventilator use, day	0.99 (0.98, 1.00)	0.050
Experience of CPR during hospitalization	1.01 (0.58., 1.75()	0.973
Concurrent anxiety disorder	1.12 (0.87, 1.44)	0.385
Concurrent depression	1.33 (1.03, 1.73)	0.031
Concurrent PTSD	2.25 (0.27, 18.42)	0.451
Concurrent alcohol abuse		
Concurrent other substance abuse		
Year of admission for ARDS		
2011 (vs 2010)	1.41 (0.82, 1.54)	0.572
2012 (vs 2010)	1.41 (0.82, 2.43)	0.210
2013 (vs 2010)	1.57 (0.93, 2.67)	0.094
2014 (vs 2010)	1.24 (0.73, 2.11)	0.421
2015 (vs 2010)	1.41 (0.84, 2.39)	0.198
2016 (vs 2010)	1.41 (0.85., 2.34)	0.179
2017 (vs 2010)	1.17 (0.69, 2.00)	0.558
2018 (vs 2010)	1.38 (0.82, 2.32)	0.226
2019 (vs 2010)	1.03 (0.59, 1.81)	0.921

Hosmer Lemeshow, chi-square: 4.88, *df* = 8, *P* = 0.770

ARDS, acute respiratory distress syndrome; OR, odds ratio; CI, confidence interval; IM, internal medicine; USD, United States Dollar; CCI, Charlson comorbidity index; ECMO, extracorporeal membrane oxygenation; NMB, neuromuscular blockade; CRRT, continuous renal replacement therapy; CPR, cardiopulmonary resuscitation; PTSD, post-traumatic stress disorder

Table 4 shows the results of the multivariable Cox regression analysis for 1-year all-cause mortality among ARDS survivors. Delirium was not associated with 1-year all-cause mortality significantly, when compared to the control group (HR 0.85, 95% CI 0.69–1.06; *P* = 0.148).

**Table 4:** Multivariable Cox regression analysis for 1-year all-cause mortality among ARDS survivors

Variable	HR (95% CI)	*P* value
Delirium group (vs control)	0.85 (0.69, 1.06)	0.148
Age, year	1.04 (1.04, 1.05)	< 0.001
Sex, male (vs female)	1.43 (1.29, 1.58)	< 0.001
Having a job at admission for ARDS	1.01 (0.91, 1.11)	0.908
Annual income level at ARDS treatment		
Q2 (vs Q1: Lowest)	0.86 (0.74, 1.01)	0.065
Q3 (vs Q1: Lowest)	0.93 (0.80, 1.07)	0.278
Q4: Highest (vs Q1: Lowest)	0.95 (0.85, 1.07)	0.411
Unknown (vs Q1: Lowest)	0.96 (0.71, 1.31)	0.814
Admitting department: IM (vs non-IM)	0.84 (0.75, 0.95)	0.005
Length of hospitalization, day	1.01 (1.00, 1.01)	< 0.001
Total cost for hospitalization, 1000 USD	1.02 (1.01, 1.02)	< 0.001
Underlying brain disability	1.25 (1.07, 1.46)	0.006
Underlying non-brain disability	1.13 (1.01, 1.26)	0.028
Annual case volume of ARDS admission		
Q2: 5–14 (vs Q1 ≤ 4)	0.85 (0.74, 0.98)	0.021
Q3: 15–28 (vs Q1 ≤ 4)	0.98 (0.86, 1.12)	0.809
Q4 ≥ 28 (vs Q1 ≤ 4)	0.93 (0.80, 1.07)	0.289
Main diagnosis of ARDS (vs Secondary diagnosis of ARDS)	0.91 (0.83, 1.00)	0.041
Sepsis associated ARDS	1.10 (0.96, 1.26)	0.168
Diagnosis of shock during hospitalization	0.99 (0.80, 1.23)	0.945
Isolated ICU admission	0.87 (0.64, 1.17)	0.346
CCI at hospital admission for ARDS		
2–3 (vs 0–1)	1.17 (1.01, 1.36)	0.043
4–5 (vs 0–1)	1.54 (1.32, 1.80)	< 0.001
≥ 6 (vs 0–1)	2.08 (1.79, 2.41)	< 0.001
ECMO support	0.66 (0.47, 0.92)	0.014
NMBA use	0.70 (0.62, 0.80)	< 0.001
CRRT use	0.90 (0.71, 1.16)	0.425
Benzodiazepine continuous infusion	1.03 (0.92, 1.15)	0.600
Opioid continuous infusion	0.75 (0.63, 0.89)	0.001
Propofol continuous infusion	0.93 (0.75, 1.16)	0.526
Duration of Mechanical Ventilator use, day	1.01 (1.01, 1.02)	< 0.001
Experience of CPR during hospitalization	1.80 (1.46, 2.22)	< 0.001
Concurrent anxiety disorder	0.89 (0.80, 0.98)	0.020
Concurrent depression	1.16 (1.05, 1.29)	0.005
Concurrent PTSD	0.41 (0.06, 2.96)	0.379
Concurrent alcohol abuse	0.89 (0.69, 1.15)	0.383
Concurrent other substance abuse	0.63 (0.28, 1.41)	0.260
Year of admission for ARDS		
2011 (vs 2010)	1.06 (0.88, 1.29)	0.524
2012 (vs 2010)	0.99 (0.82, 1.21)	0.928
2013 (vs 2010)	1.13 (0.93, 1.38)	0.227
2014 (vs 2010)	0.94 (0.77, 1.14)	0.522
2015 (vs 2010)	0.97 (0.80, 1.17)	0.739
2016 (vs 2010)	0.97 (0.80, 1.17)	0.720
2017 (vs 2010)	0.88 (0.72, 1.08)	0.214
2018 (vs 2010)	0.91 (0.74, 1.12)	0.395
2019 (vs 2010)	0.80 (0.64, 0.99)	0.039

ARDS, acute respiratory distress syndrome; HR, hazard ratio; CI, confidence interval; IM, internal medicine; USD, United States Dollar; CCI, Charlson comorbidity index; ECMO, extracorporeal membrane oxygenation; NMB, neuromuscular blockade; CRRT, continuous renal replacement therapy; CPR, cardiopulmonary resuscitation; PTSD, post-traumatic stress disorder

## Discussion

In this population-based cohort study in South Korea, 4.7% of ARDS survivors were diagnosed with delirium during hospitalization. Male sex, longer duration of hospitalization, neuromuscular blockade use, benzodiazepine and propofol continuous infusion, and concurrent depression were potential risk factors for the occurrence of delirium in patients with ARDS. However, the occurrence of delirium was not associated with the 1-year all-cause mortality in this study. Our results suggest that although ARDS survivors experienced delirium during hospitalization, delirium did not affect the relative long-term survival after hospital discharge.

A recent cohort study by Kalra et al. reported that 43% (124/286) of patients with ARDS were diagnosed with ICU delirium [12], which is a much higher prevalence than that in our study. Several reasons might explain this difference. First, we included ARDS survivors who were alive for ≥ 60 days after ARDS diagnosis, while Kalra et al. included all patients diagnosed with ARDS using the Berlin definition in their study [12]. Patients with ARDS who were diagnosed with delirium and died within 60 days after the diagnosis of delirium were excluded from our study. Second, the CAM-ICU assessment tool was used to define delirium in the study by Kalra et al. [12], whereas we used registered ICD-10 codes in a large population. Therefore, some cases may have been missed in our study due to variations in delirium diagnosis in each hospital in South Korea.

Male sex has been a known risk factor for delirium in hospitalized elderly patients [21, 22]. Previous studies have reported that male sex is also a risk factor for the development of delirium after hip fracture surgery [23] and vascular surgery [24]. However, in a recent review, sex was not associated with the risk of delirium in adult ICU patients [25]. Although the biological mechanism of the relationship between sex and delirium remains unclear, sex differences in immune responses to stressors may explain this mechanism [26, 27]. Inflammation plays a major role in the pathogenesis of delirium [28]. A previous study reported that men had a higher C-reactive protein response to an endotoxin challenge than women [26]. However, another previous study reported contradictory findings: pro-inflammatory and innate immune responses were higher among women [27]. Therefore, the effect of sex on the development of delirium in critically ill patients such as patients with ARDS needs to be confirmed in future studies.

In our study, concurrent ARDS was a risk factor for the development of delirium among ARDS survivors; this was consistent with the results of the study by Kalra et al. [12]. Concurrent depression is an important risk factor of delirium among elderly and hospitalized patients [29]. Moreover, concurrent depression is known to be interrelated with delirium and to have a clinical overlap with delirium among elderly people [18]. However, in our study, the concurrent anxiety disorder and PTSD were not associated with the development of delirium among ARDS survivors. Meanwhile, Kalra et al. reported that concurrent anxiety disorder was associated with delirium in patients with ARDS [12]. Thus, future studies on the risk factors of delirium among patients with ARDS should consider the various types of mental illness.

The increased association of sedative use (benzodiazepine or propofol) with delirium in patients with ARDS was an important finding. Although patients with ARDS usually require deep sedation for mechanical ventilation, the protocol-based light sedation strategies had been suggested based on previous reports [30]. Hager et al. reported that reducing deep sedation was associated with decreased delirium in patients with acute lung injury [31]. Therefore, the deep sedation using propofol or benzodiazpine might increase risk of delirium in patients with ARDS in studies. Moreover, it was also possible that patients who received deep sedation using propofol or benzodiazepine might be more severe than patients with ARDS, and the risk of delirium was higher than other patients with ARDS. As the relationship between depth of sedation and risk of delirium among mechanically ventilated patients remains controversial and inconclusive issue [32], more research is needed regarding this issue.

Interestingly, there was no significant association between the occurrence of delirium during hospitalization and 1-year mortality among ARDS survivors in this study. There are previous studies on the association between the occurrence of delirium and 1-year mortality [4, 33–35]. Pisani et al. reported that the duration of delirium was associated with increased 1-year all-cause mortality in older ICU patients [4]. Contrarily, Wolters et al. reported that delirium during ICU stay was not associated with 1-year mortality among survivors of a critical illness in the Netherlands [36], which is similar to the results of our study. The varying characteristics of the study populations (overall ICU population and survivors) of the two studies might explain the differences [4, 36]. Wolters et al. excluded ICU patients who died during their ICU stay [36], and this was consistent with our study. In contrast, Pisani et al. did not exclude ICU patients who died during their ICU stay or hospitalization [4]. The occurrence of delirium during hospitalization has been reported to increase hospital mortality among critically ill patients [37]; therefore, we excluded patients with ARDS who had more severe conditions, who might have been diagnosed with delirium, and died during hospitalization. The exclusion of the patients who died within 60 days of ARDS diagnosis may have influenced the results.

This study had several limitations. First, the severity of ARDS was not assessed accurately. For example, the PaO2/FiO2 ratio (ratio of the patient's oxygen in arterial blood [PaO2] to the fraction of oxygen in inspired air [FiO2]) and Acute Physiology and Chronic Health Evaluation II scores were not considered in this study for accurate adjustment of ARDS severity. Second, some treatment information, such as prone positioning, was not considered in this study because there is no prescription code for prone positioning in South Korea. Third, the model was not adjusted for important variables such as smoking history, pulmonary function test results, and body mass index. This is because their data were unavailable in the NHIS database. Lastly, the CAM-ICU, which is known to be the best measuring tool for the evaluation of delirium, was not used in this study due to unavailable data in the NHIS database. Therefore, some cases may have been missed or inaccurate diagnoses of delirium may have been made: these may also have affected the results. Moreover, we cannot guarantee that all patients with delirium were diagnosed and treated using the DSM-5 criteria in this study [2]. In these perspectives, the methodology using ICD-10 code of delirium made our study include obvious and serious cases that required immediate treatment of delirium. This limitation regarding methodology using ICD-10 code should be considered the prevalence of delirium and associated factor in this study.

## Conclusions

In conclusion, 4.7% of ARDS survivors were diagnosed with delirium during hospitalization in South Korea. Male sex, longer duration of hospitalization, neuromuscular blockade use, benzodiazepine and propofol continuous infusion, and concurrent depression were potential risk factors for the development of delirium, but the occurrence of delirium might not affect 1-year all-cause mortality among ARDS survivors.

## Supplementary Information

**Additional file 1 Table S1.** The ICD-10 codes used by comorbidity to compute the Charlson comorbidity index

## Data Availability

All data will be available upon reasonable request to corresponding author.

## Abbreviations

ARDS: Acute respiratory distress syndrome
CAM-ICU: Confusion assessment method for the ICU
CCI: Charlson comorbidity index
CI: Confidence intervals
DSM-5: Diagnostic and statistical manual of mental disorders, fifth edition
HR: Hazard ratios
ICU: Intensive care unit
IRB: Institutional Review Board
OR: Odds ratios
SD: Standard deviation
